# Foot and ankle characteristics and dynamic knee valgus in individuals with patellofemoral osteoarthritis

**DOI:** 10.1186/s13047-018-0310-1

**Published:** 2018-12-11

**Authors:** Narelle Wyndow, Natalie J. Collins, Bill Vicenzino, Kylie Tucker, Kay M. Crossley

**Affiliations:** 1 School of Health & Rehabilitation Sciences, The University of Queensland, St Lucia, Queensland 4072 Australia; 2 La Trobe Sport and Exercise Medicine Research Centre, College of Science, Health and Engineering, La Trobe University, Bundoora, Victoria 3086 Australia; 3 School of Biomedical Sciences, The University of Queensland, St Lucia, Queensland 17 4072 Australia

## Abstract

**Study design:**

Controlled laboratory study; cross-sectional design.

**Background:**

Foot and ankle characteristics and dynamic knee valgus differ in people with and without patellofemoral (PF) pain. However, it is unknown if these characteristics are evident in people with PF osteoarthritis (OA), compared to pain-free older adults.

**Objectives:**

To compare foot and ankle mobility, foot posture and dynamic knee valgus, measured as the frontal plane projection angle (FPPA) during single-leg squatting, between individuals with and without PFOA.

**Methods:**

Fifty-one participants with PFOA (66% women, mean ± SD age 57 ± 10 years, body mass index (BMI) 27 ± 6 kg/m^2^), and 23 controls (56% women, age 56 ± 9 years, BMI 24 ± 4 kg/m^2^) had ankle dorsiflexion measured using the knee-to-wall test, foot mobility calculated as the difference in midfoot height or width between non-weightbearing and weightbearing, and static foot posture characterized utilizing the Foot Posture Index. Peak FPPA was determined from video recordings while participants performed 5 single-leg squats. Linear regressions examined between-groups relationships for foot and ankle characteristics and the FPPA.

**Results:**

The PFOA group had less ankle dorsiflexion (odds ratio 6.7, 95% confidence interval 2.46–18.2), greater midfoot height mobility (5.2, 1.78–15.14) and width mobility (4.3, 1.33–14.39), and greater foot mobility magnitude (8.4, 2.32–30.69) than controls. There was no difference in FPPA (knee valgus angle) between groups (15, 0.63–377.99).

**Conclusion:**

Foot and ankle characteristics were different in individuals with PFOA compared to control participants, however there was no difference in dynamic knee valgus during single leg squat. Clinical interventions to address greater foot mobility may be relevant for PFOA.

## Introduction

The patellofemoral (PF) joint is affected by osteoarthritis (OA) in approximately 50% of people with knee pain or radiographic OA [1], and PFOA is associated with considerable pain and functional limitations [2]. The clinical symptoms of PFOA, such as anterior knee pain during stair ambulation and squatting, share many similarities with PF pain in adolescents and young adults. PFOA is clinically differentiated from PF pain based on imaging evidence of cartilage or osseous changes, and it has been proposed that the two conditions may form a disease continuum [3]. If so, it is possible that people with PFOA and PF pain could share similar structural and functional features [3].

Patellofemoral pain is generally considered to result from heightened PF loads, related to aberrant lower limb biomechanics [4]. Dynamic knee valgus during the single-leg squat can be measured as the frontal plane projection angle (FPPA) of the knee, and results from a combination of hip internal rotation, hip adduction, and knee flexion and abduction [5]. Greater FPPA is seen in people with PF pain [6] and is thought to contribute to altered joint loads in this population [3, 6]. It is unknown if the FPPA is greater in those with PFOA compared to people without PFOA, and hence a potential contributor to PFOA symptoms.

Greater midfoot mobility, and lesser ankle joint dorsiflexion range are evident in some, but not all people with PF pain compared to healthy individuals [4]. Although the direct relationship between foot and ankle characteristics and PF loads are unknown, greater foot mobility and lower ankle dorsiflexion are associated with greater FPPA [4, 5]. Our prior work demonstrated that midfoot mobility in people with PF pain gradually decreases between 18 and 50 years of age, with significantly lower midfoot mobility observed in those > 40 years compared to those between 18 and 29 years of age [7]. However, it is not known whether people with PFOA demonstrate differences in foot mobility when compared to age-matched pain-free controls. If those with PFOA have greater midfoot mobility or lower ankle dorsiflexion range, further investigation into the role of the foot and ankle in PFOA aetiology and management may be warranted.

The primary aim of this study was to determine if individuals with PFOA demonstrate differences in the FPPA of the knee as well as foot and ankle characteristics, compared to age-matched controls.

## Methods

This cross-sectional study was approved by The University of Queensland’s Medical Research Ethics Committee (approval number: 2014000068). PFOA participants were recruited into a phase II, randomized controlled trial, investigating the effect of prescribed footwear and foot orthoses compared to prescribed footwear alone in PFOA (ACTRN12615000002583). Data used in the current study was collected at baseline, prior to randomization.

### Participants

Volunteers were recruited from advertisements on social media, seniors’ newsletters and staff newsletters at The University of Queensland. Inclusion and exclusion criteria are detailed in Table 1.

**Table 1:** Inclusion and Exclusion for the Control and PF OA Groups

Inclusion Criteria Both Groups	
(i) aged ≥40 years;	
Exclusion Criteria Both Groups	
(i) current or previous pain in the knee, hip, lumbar spine or foot that had lasted longer than 3 months and/or required intervention; (ii) foot orthoses use in the last 12 months. (iii) a history of hip, knee or foot surgery; (iv) neurological or systemic arthritis conditions; (v) planned lower limb surgery in the following 2 months; (vi) physical inability to undertake testing procedures; (vii) an inability to understand written and spoken English.	
Specific Inclusion Criteria PF OA	
(i) anterior knee pain aggravated by at least two activities that load the PF joint (e.g. squatting, stair ambulation); (ii) pain during these activities present on most days in the past month; (iii) pain severity ≥30 mm on a 100 mm visual analogue scale during aggravating activities; (iv) radiographic evidence of PF OA (Kellgren and Lawrence ≥ grade 1 [19]).	
Specific Exclusion Criteria PF OA	
(i) concomitant pain from other knee structures (including the TF joint), hip or lumbar spine; (ii) recent treatment for PF pain (knee injections within the previous 3 months; (iii) foot orthoses or physiotherapy within the previous 12 months) (iv) moderate to severe concomitant TF OA (Kellgren and Lawrence grade ≥ 3 on radiograph); (v) contraindications to x-ray.	

### Procedures

Each participant gave written informed consent prior to participation. Participant characteristics (age, height, weight, sex) were collected at baseline.

For foot and ankle mobility and posture, and single-leg squat assessments, participants were barefoot and wore brief shorts. Both legs were tested in a random order. Details of the methods have been reported previously [5], but are briefly described below.

### Ankle and foot mobility and posture measures

#### Ankle mobility

To measure ankle dorsiflexion range, the knee-to-wall lunge method was used [5]. The distance from the longest toe to the wall, whilst maintaining the heel in contact with the ground and the foot aligned in the sagittal plane, was recorded in centimeters. This method has demonstrated excellent reliability and is highly correlated with tibial angle measures of ankle dorsiflexion (*r* > 0.93) [8].

#### Foot mobility

**Fig. 1:**
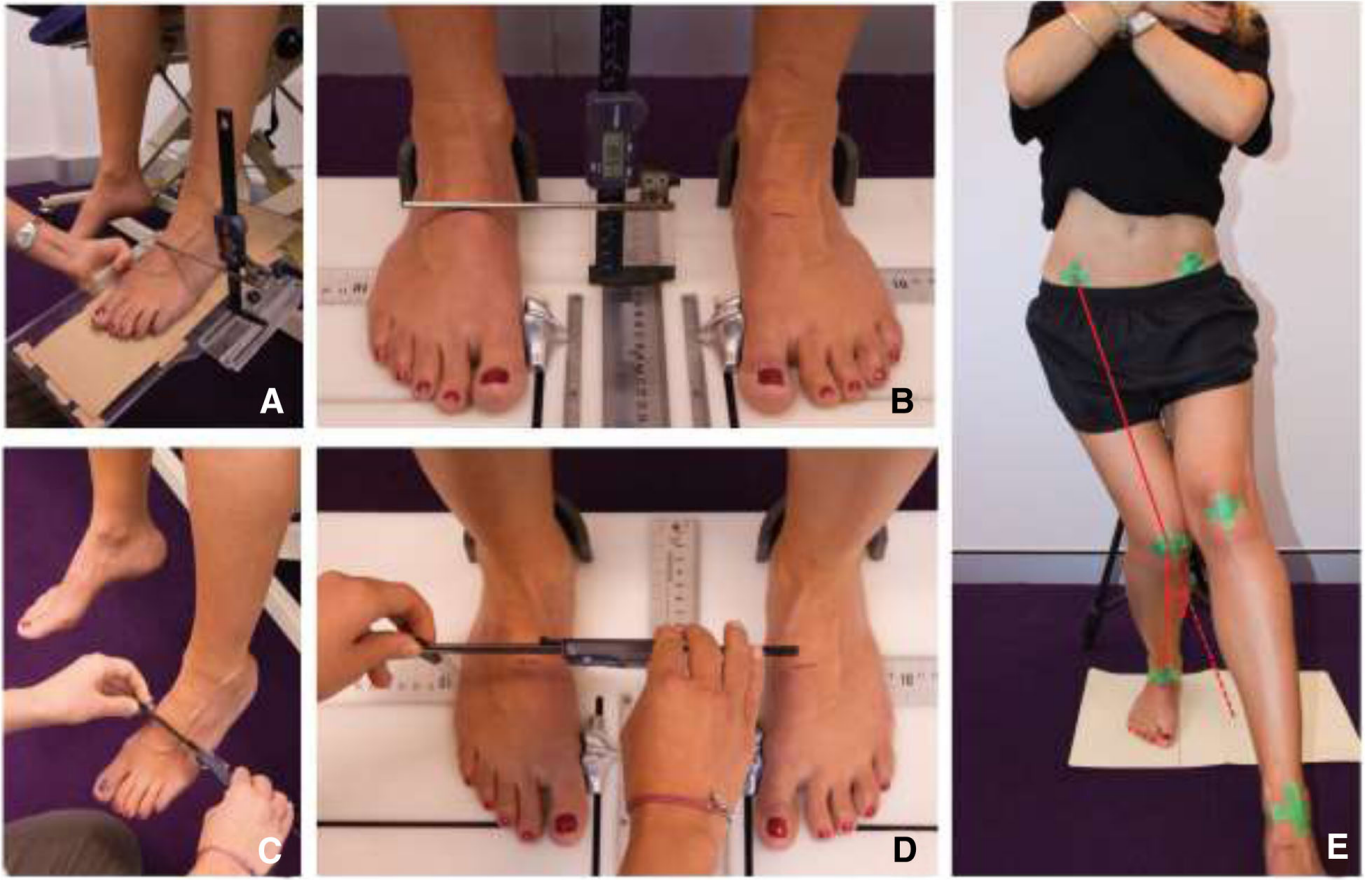
Foot mobility and frontal plane projection angle measurements. Measurement of arch height non-weightbearing (**a**), and weightbearing (**b**). Measurement of midfoot width non-weight bearing (**c**) and weight bearing (**d**). Measurement of the frontal plane projection angle at the deepest point of the single-leg squat (**e**)

Foot mobility was quantified using methods with established reliability [9]. Briefly, midfoot height and width were measured at 50% of total foot length using digital calipers [9]. Foot mobility was characterized by: (i) the difference in midfoot height from non-weight bearing to standing (Fig. 1a and b) and; (ii) the difference in midfoot width from non-weight bearing to standing (Fig. 1c and d). The foot mobility magnitude, a composite measure of sagittal and mediolateral arch mobility, was calculated as √((midfoot arch mobility)^2^ + (midfoot width mobility)^2^).

#### Static foot posture

Static foot posture in bilateral stance was quantified with the Foot Posture Index (FPI) as in our previous study [5]. The FPI rates static foot posture in 3 planes to give a composite score ranging from − 12 (maximally supinated) to + 12 (maximally pronated).

#### Frontal plane projection angle measurement

Colored markers were placed bilaterally over the anterior superior iliac spines, the midpoint of the femoral condyles, and the midpoint of the malleoli. Digital video recordings (PAL 25 frames per second, resolution 800 × 600 pixels, Nikon Coolpix AW110, Nikon, Tokyo, Japan) of the frontal plane were made while participants performed the single-leg squat test.

Participants stood with their feet aligned in the sagittal plane, indicated by markings on the floor, and their arms folded across their chest (Fig. 1e). This standardized testing position was utilized to minimize the impact of compensatory strategies on single-leg squat performance. Participants were instructed to squat until their buttocks touched a tripod positioned behind them, then immediately rise to the starting position. Participants performed five continuous repetitions on each leg, to a depth of 45°, at a cadence of 2 seconds per rise and 2 seconds per lower as indicated by a metronome [5].

Still frames taken from the raw video footage (QuickTime Player v10.4, Apple Inc.) of the deepest part of the squat, as indicated by the knee marker (peak knee flexion), were imported into an image-processing program (ImageJ, Public Domain, National Institutes of Health). Lines were superimposed to connect the hip, knee and ankle markers for the calculation of the angle formed at the knee (FPPA) in degrees (Fig. 1e). A varus knee angle was defined as a negative deviation from a neutral alignment of 180 degrees, whereas a valgus knee angle was defined as a positive deviation from a neutral alignment of 180 degrees. Three out of the five single-leg squat trials were used for analysis. The trials were selected based on overall performance (maintenance of balance, achieving the correct depth of squat and visibility of anatomical markers). If all trials were performed correctly, the average of the middle 3 trials were utilsed.

#### Statistical analysis

Between-group differences for participant characteristics were evaluated using independent t-tests, while the differences in foot and ankle characteristics and the FPPA were evaluated using linear regression models with generalized estimating equations (GEEs). Utilizing GEEs allowed for inclusion of bilateral lower limb data, as most participants with PFOA had bilateral pain (61%) [10]. Participant group was entered as a factor, while sex, height and weight were entered as covariates.

Odds ratios (OR) with 95% confidence intervals (CI) were generated for each model. Significance was set at 0.05. All analyses were performed with IBM SPSS version 23 (Chicago Illinois, USA). Data were sign converted to remove negative Beta values for ease of interpretation.

## Results

Fifty-one individuals with PFOA (34 [66%] women, mean ± SD age 57 ± 10 years, body mass index (BMI) 27 ± 6 kg/m^2^), and 23 controls (13 [56%] women, age 56 ± 9 years, BMI 24 ± 4 kg/m^2^) participated (Table 2). Those with PFOA were heavier and shorter than those in the control group (p = < 0.05). For the single-leg squat task, data were not collected for 7 (13%) participants with PFOA due to an inability of these individuals to complete the task (due to knee pain) or an inability to visualize the hip marker due to excessive forward trunk flexion during the task.

**Table 2:** Participant Characteristics and Between-Group Differences

	PF OA *n* = 51	Controls *n* = 23				
	Mean (SD)	Mean (SD)	Mean Diff	Beta (95% CI)	*p* values	OR (95% CI)
Gender % female	66%	56%	10			
Age	57 (10)	56 (9)	1		0.89	
Height (m)	1.68 (10)	1.72 (11)	−4		0.12	
Weight	78 (18)	72 (16)	6		0.17	
BMI	27 (6)	24 (4)	4		0.08	
Ankle DF (cm)	9 (3)	11 (3)	−2	1.90(0.9–2.9)	0.0001**	6.7 (2.5–18.2)
Midfoot height mobility (mm)	14 (3)	12 (3)	2	1.64(0.6–2.7)	0.002*	5.2 (1.8–15.1)
Midfoot width mobility (mm)	13 (3)	11 (3)	2	1.47(0.3–2.6)	0.015*	4.3(1.3–14.4)
Foot Mobility Magnitude (mm)	19 (3)	17 (4)	2	2.13(0.8–3.4)	0.001**	8.45(2.3–30.7)
Foot Posture Index	4 (3)	3 (3)	1	0.90(−.1–1.9)	0.086	2.48(0.9–6.9)
FPPApeak (°)*PF OA *n* = 44	8 (8)	6 (9)	2	2.75(−0.5–5.9)	0.09	15.41(0.6–378.0)

Those with PFOA had less ankle dorsiflexion (OR: 6.7 95%CI: 2.46–18.2), greater midfoot height mobility (OR: 5.2; 95%CI 1.78–15.14), greater midfoot width mobility (OR: 4.3; 95%CI: 1.33–14.39), and greater foot mobility magnitude (OR: 8.45; 95%CI: 2.32–30.69) compared to controls. The PFOA group demonstrated no significant difference in FPPA (knee valgus angle) compared to the control group (OR: 15.41; 95%CI: 0.63–377.99; *p* = 0.09).

## Discussion

Individuals with PFOA had small, but significant, differences in foot and ankle mobility and foot posture, compared to age-matched controls. However, the FPPA (dynamic knee valgus) was not significantly different between groups.

Individuals with PFOA had 2 cm less ankle dorsiflexion range than their age-matched, pain-free controls; a difference 4 times greater than the standard error of the measurement (SEM) (SEM: 0.5 cm) [8]. The significance of less static ankle dorsiflexion range in those with PFOA is unclear. Younger populations with PF pain appear to have greater static ankle dorsiflexion measures than controls [11], however findings are more variable in those with PF pain in the third decade [12, 13]. No studies have compared peak dynamic ankle dorsiflexion in older adults with and without PF pain or OA. Further studies exploring associations between static ankle range and dynamic ankle excursion across different age groups, and with PF symptomatic and structural severity may assist in our understanding of the relevance of our findings.

Midfoot mobility was greater in people with PFOA. While the differences between the PFOA group and pain-free group were small (foot mobility magnitude 2.1 mm), they were greater than the SEM (foot mobility magnitude SEM 1.3 mm [9]). This is in agreement with findings in younger people with PF pain (mean age 29 years) who had 2 mm greater foot mobility magnitude than age-matched controls (PF pain: 18 mm; controls: 16 mm) [14]. However, the relationship between midfoot mobility and PF loads in unknown. Higher midfoot mobility is likely to exert its greatest functional impact on the lower limb in the second half of stance phase (from midstance to toe-off). While no studies have evaluated the relationship between foot mobility, knee kinematics and knee loads at this time, individuals with PFOA experience higher knee flexion moments, impulse and PF joint stress in the second half of stance phase than healthy controls [15]. Since interventions such as foot orthoses, which aim to support the midfoot, have known efficacy for improving PF pain in younger individuals with high midfoot mobility [16], a similar response to foot orthoses may be observed in people with PFOA.

Dynamic valgus, measured as the FPPA, was not significantly greater in the PFOA group than the control group. The 2° between-group difference is half of the difference observed in people with PF pain compared to healthy controls (4°, mean age 23 years) [6], and less than that of the SEM (2.8° [17]). The control group in this current study demonstrated an average FPPA of 6°, compared to 3° in the PF pain cohort of Willson et al. [6]. The degree of FPPA in our PFOA group was similar to that seen in PF pain (PFOA: 8°, PF pain: 7°) [6]. Poor performance on single-leg squat in healthy middle-aged adults is common (40–60 years of age) and may reflect age-related balance deterioration [18]. Thus, based on the current data, an increased FPPA does not appear to be a useful clinical tool to differentiate those with or without PFOA in people over 40 years of age.

### Clinical implications

Our findings may have potential clinical implications for people with PFOA. Foot mobility was greater in people with PFOA than age-matched controls. The magnitude of the between-group differences in midfoot mobility were similar to those observed in younger people with PF pain [14]. Thus, interventions to address greater foot mobility in people with PFOA, such as foot orthoses, footwear or foot strengthening, may provide clinical benefits based on positive outcomes in people with PF pain [16].

The clinical implications of the lower ankle dorsiflexion range observed in the PFOA group warrants clinical consideration based on its potential impact on sagittal plane PF joint loading. However, as the reduction in ankle range may be related to active pain-minimizing compensatory strategies, it is uncertain whether common clinical interventions such as calf stretching and heel lifts, would be effective at increasing active sagittal plane ankle range in PFOA. Movement retraining strategies, coupled with treatments aimed at reducing knee pain (e.g. patellar taping) may be more effective in some individuals. The effect of these interventions on PF loads and pain in PFOA needs further investigation.

## Conclusion

In conclusion, this study identified lower ankle dorsiflexion range and greater foot mobility in those with PFOA compared to age-matched controls. Consideration of clinical interventions to reduce the potential impact of increased foot mobility are warranted in people with PFOA.

### Significance and innovations: (2–4 bullet points)

Findings:

- Individuals with PFOA have lower ankle dorsiflexion range and greater midfoot mobility than age-matched pain-free controls.

Implications:

- Interventions to reduce midfoot mobility warrant investigation in people with PFOA.

Cautions:

- The degree of FPPA on single-leg squat is unable to differentiate those with and without PFOA in older populations.

## Abbreviations

BMI: Body mass index
CI: Confidence interval
cm: Centimeter
FPPA: Frontal plane projection angle
GEE: Generalized estimating equations
m: Meter
mm: Millimeter
OA: Osteoarthritis
OR: Odds ratio
PF: Patellofemoral
PFOA: Patellofemoral osteoarthritis
SD: Standard deviation
SEM: Standard error of the measurement
TF: Tibiofemoral
